# Divergence and introgression among the *virilis* group of *Drosophila*

**DOI:** 10.1002/evl3.301

**Published:** 2022-11-28

**Authors:** Leeban H. Yusuf, Venera Tyukmaeva, Anneli Hoikkala, Michael G. Ritchie

**Affiliations:** ^1^ Centre for Biological Diversity, School of Biology University of St Andrews St Andrews KY16 9TH United Kingdom; ^2^ Department of Evolution, Ecology and Behaviour University of Liverpool Liverpool L69 7ZB United Kingdom; ^3^ Department of Biological and Environmental Science University of Jyväskylä Jyväskylä 40014 Finland

**Keywords:** Divergence, gene flow, introgression, phylogenomics, reproductive isolation, speciation

## Abstract

Speciation with gene flow is now widely regarded as common. However, the frequency of introgression between recently diverged species and the evolutionary consequences of gene flow are still poorly understood. The *virilis* group of *Drosophila* contains 12 species that are geographically widespread and show varying levels of prezygotic and postzygotic isolation. Here, we use de novo genome assemblies and whole‐genome sequencing data to resolve phylogenetic relationships and describe patterns of introgression and divergence across the group. We suggest that the *virilis* group consists of three, rather than the traditional two, subgroups. Some genes undergoing rapid sequence divergence across the group were involved in chemical communication and desiccation tolerance, and may be related to the evolution of sexual isolation and adaptation. We found evidence of pervasive phylogenetic discordance caused by ancient introgression events between distant lineages within the group, and more recent gene flow between closely related species. When assessing patterns of genome‐wide divergence in species pairs across the group, we found no consistent genomic evidence of a disproportionate role for the X chromosome as has been found in other systems. Our results show how ancient and recent introgressions confuse phylogenetic reconstruction, but may play an important role during early radiation of a group.

Two major themes emerging from the rise of genomic approaches to phylogenetics and speciation are an understanding that genomic divergence is usually extremely patchy and that contemporary or historical introgression between species can be extensive (Nosil et al. 2009; Suvorov et al. 2022). Heterogeneous genomic divergence has multiple potential causes. Genomic structure, especially inversions and other causes of variation in recombination rate, is associated with species’ divergence rates (Cruickshank and Hahn 2014; Wolf and Ellegren 2017). Natural and/or sexual selection may act locally on areas of genomes that therefore diverge more rapidly than the general “background” genomic divergence rate. Divergent regions probably contain barrier loci that contribute to adaptation and reproductive isolation, but genomic structure, demography, and drift also affect patterns of divergence (Ravinet et al. 2017). Gene flow following hybridization may reduce divergence in regions of the genome that introgress between diverging species, and substantial proportions of such regions may be shared between species. There are numerous examples of introgression between recent species, including during adaptive radiations (McGee et al. 2020).

The most notable example of gene flow between closely related species is in hominins, where 1%–3% of admixed proportion of DNA sequence in Eurasian populations results from introgression from Neanderthals (Sankararaman et al. 2016, 2014; Vernot and Akey 2014). Studies of a range of species including *Heliconius* butterflies (Nadeau et al. 2013; Zhang et al. 2016; Edelman et al. 2019), African cichlids (Meier et al. 2017; Malinsky et al. 2018; Svardal et al. 2020), *Solanum* (Pease et al. 2016), Darwin's finches (Lamichhaney et al. 2015, 2018; Han et al. 2017), and *Anopheles* (Fontaine et al. 2015; Thawornwattana et al. 2018; Small et al. 2020) all show evidence of substantial introgression. This may be more likely during recent divergence, but in dire wolves (Perri et al. 2021), introgression has occurred during early divergence and is absent between more recent, isolated species. We do not know the extent to which hybridization contributes to patchy genomic divergence, but it would need to be extensive to cause background homogenization of the genome (as early verbal models suggested). Some chromosomes may be more resistant to introgression. Sex chromosomes often have disproportionate effects on reproductive isolation, so gene flow might be reduced on sex chromosomes if they contain more barrier loci, resulting in higher levels of divergence (Ellegren et al. 2012; Wolf and Ellegren 2017). However, there are multiple reasons why sex chromosomes often show rapid divergence between species, including demographic effects and more effective background selection (Charlesworth et al. 1987).

The extent of introgression between species has revised our understanding of how gene flow may influence speciation. It has long been thought that gene flow will restrict divergence between species, except in regions maintained by strong selection (Barton and Bengtsson 1986; Wu and Ting 2004). However, gene flow between lineages could also facilitate speciation by acting as a conduit for adaptive genetic variation (Marques et al. 2019b). This “combinatorial” view of speciation proposes that ancestral genetic variation can be reshuffled into unique combinations that may be favored by ecological or other selection, circumventing the need for a build‐up of de novo mutations. Long recognized in polyploid plants (Abbott et al. 2013), this has also been seen to occur much more extensively during homoploid speciation, including animals (Marques et al. 2019b). Evidence of selection acting on introgressed genomic regions has been implicated in the maintenance of ecological barriers in *Heliconius* butterflies, Darwin's finches, cichlids, and sticklebacks (Nadeau et al. 2013; Marques et al. 2016, 2019a; Zhang et al. 2016; Han et al. 2017; Meier et al. 2017; Samuk et al. 2017; Lamichhaney et al. 2018; Malinsky et al. 2018; Nelson and Cresko 2018; Svardal et al. 2020). However, accurately quantifying the amount of introgression, adaptive or otherwise, during clade divergence remains an important challenge.

Studies of *Drosophila* have been important in our understanding of genomic divergence and introgression, and such studies consist of both broad scale studies and detailed analyses of species groups (Lohse et al. 2015; Mai et al. 2020; Korunes et al. 2021). Genome scans have provided evidence for two patterns: inversions contribute disproportionately to genome‐wide divergence, and introgression is lower on the X chromosome compared to the autosomes, as predicted (Garrigan et al. 2012; Turissini and Matute 2017; Schrider et al. 2018). In the *simulans* species complex, gene flow is extensive with 2.9%–4.6% of the genome showing introgression between *Drosophila sechellia* and *Drosophila simulans*. These include regions demonstrating selective sweeps suggesting adaptive introgression of genes involved in chemical perception (Garrigan et al. 2012; Brand et al. 2013; Schrider et al. 2018). Conversely, Turissini and Matute (2017) found minimal, older introgression between species in the *Drosophila yakuba* clade. Recently, a broad study of over 150 species of *Drosophila* concluded that both ancient and more contemporary introgressions were widespread, occurring in most clades, and could regularly involve >10% of the genome (Suvorov et al. 2022). However, the chromosomal location and identity of introgressed regions were not mapped.

Here, we use new and existing sequence data from the *virilis* species group of *Drosophila* to examine genetic divergence and gene flow. We have three main objectives: (1) to produce an accurate estimate of the phylogeny of the group, (2) to examine levels of divergence across autosomes and sex chromosomes, and (3) to examine the extent and genomic location of introgression across the group. Historically, the *virilis* group was thought to consist of 13 species that belong to two “phylads” or subgroups, the *montana* (usually thought to contain eight species) or *virilis* phylads (five species) (Throckmorton 1982). Species in the *virilis* group typically inhabit temperate ancient forest regions, with the exception of *Drosophila virilis* that is cosmopolitan and inhabits timber yards, breweries, and market places (Patterson 1952; Throckmorton 1982). The group is thought to have originated in East Asia and spread into North America via Beringia, and there are members of each phylad in the Nearctic and Palearctic regions (Throckmorton 1982). Morphological classification and molecular phylogenetics have failed to clarify the evolutionary relations of the group, especially the deeper branches (Patterson 1952; Chekunova et al. 2008; Morales‐Hojas et al. 2011).

The *virilis* group has been used extensively to study adaptation and speciation (Throckmorton 1982; Hoikkala and Poikela 2022). Postzygotic barriers have evolved to varying degrees (Throckmorton 1982; Orr and Coyne 1989) and postmating prezygotic (PMPZ) barriers have been shown to cause reductions in both interspecific and interpopulation fertilization (Sweigart 2010; Ahmed‐Braimah 2016; Garlovsky and Snook 2018; Poikela et al. 2019; Garlovsky et al. 2020). Species also show strong sexual isolation influenced by male courtship song and/or cuticular hydrocarbons (Hoikkala and Lumme 1987; Liimatainen and Hoikkala 1998; Ritchie et al. 2001; Poikela et al. 2019). Morphological differences, particularly in body pigmentation, have been described, although the evolutionary processes underpinning pigmentation differences are not well understood (Wittkopp et al. 2002a, 2002b; Kulikov et al. 2004; Bubliy et al. 2007; Ahmed‐Braimah and Sweigart 2015; Lamb et al. 2020). Several species of the *virilis* group, especially those of the *montana* phylad, persist in extreme cold environments, and show high cold acclimation and diapause that may contribute to genomic divergence (Vesala et al. 2012; Parker et al. 2016, 2015; Salminen et al. 2015; Tyukmaeva et al. 2015; Wiberg et al. 2021). Fixed and polymorphic chromosomal inversions have been reported within the group and may be driven by the activity and expansion of transposable elements, giving rise to regions of high differentiation between species and populations (Evgen'ev et al. 2000; Fonseca et al. 2013; Reis et al. 2018).

Here, we use new whole‐genomic sequence data from the *virilis* group to resolve their phylogeny and confirm that the major clade split is old and probably occurred in Miocene. We also show that gene flow has been extensive between some lineages despite the relatively rapid evolution of multiple sources of reproductive isolation between populations and species.

## Methods

We obtained new sequences of 15 individual flies of nine species (see Table S1) and supplemented these with available sequences to obtain data for all species except *Drosophila texana*. We constructed timed phylogenies, examined rates of evolution, and tested for introgression using methods described in detail in the Supporting Information. We also obtained published data on reproductive isolation within the group and compared this with inferred levels of genetic divergence. See the Supporting Information for full details.

## Results

### GENOME ASSEMBLY

Altogether, we assembled 15 new genomes, with at least one for each species in the *D. virilis* group, with the exception of *D. texana*. We added published genomes for *Drosophila americana* (Fonseca et al. 2013), *D. virilis* (Flybase version 1.07), and (as an outgroup) *Drosophila mojavensis* (Flybase version 1.04). Genome size for the assembled genomes ranges from 170 to 210 MB, which corresponds to the range of published genomes for the group. The new assemblies show relatively high completeness (>90%) using the BUSCO Diptera reference gene set (Table S1). We identified 6%–8% of the assemblies as repeat content, apart from *Drosophila ezoana* that showed higher levels (17%). With the exception of our annotation for *Drosophila lacicola*, we found between 13,100 and 18,075 genes in the genome assemblies, consistent with other *Drosophila* genome annotations. Finally, we characterized rates of molecular evolution in protein‐coding genes and identified rapidly evolving genes in the *virilis* group (see Supporting Information).

### PHYLOGENETIC RECONSTRUCTION AND DATING

**Figure 1:**
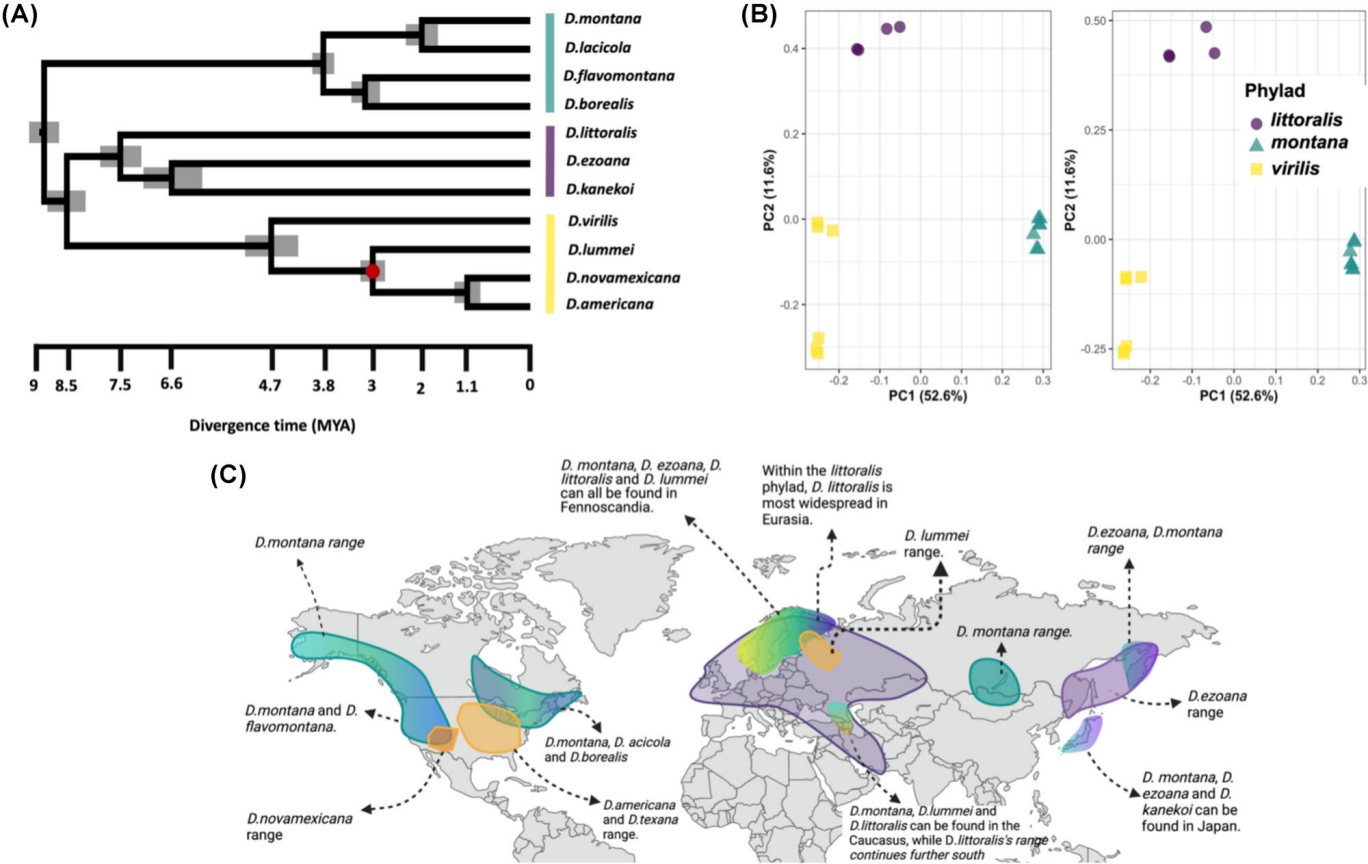
Species tree of the *virilis* group with estimated divergence times. (a) Species tree reconstruction was performed using ASTRAL and gene trees for 1336 single‐copy orthologs. Divergence times were estimated using the BPP program (see Supplementary material) and randomly sampled, genome‐wide small introns (100 loci consisting of 75–85 bp small introns). Posterior estimates for divergence times were scaled using a single calibration point (denoted by the red dot; see text for details). (b) Principal component analysis (PCA) showing species relationships using randomly sampled SNPs on the X chromosome (3218 SNPs) and autosomes (12,272 SNPs). (c) Map showing putative ranges for species in all three phylads. Created with BioRender.com

We found agreement between phylogenies produced by maximum likelihood and species tree reconstruction. All relationships within both concatenated maximum‐likelihood phylogeny and species tree were recovered with maximal (100%) bootstrap support. Our phylogeny is broadly consistent with previous phylogenetic reconstructions of the *virilis* group (Wang et al. 2006; Chekunova et al. 2008; Morales‐Hojas et al. 2011), although we have better resolved the earlier branches. In previous phylogenies (Orsini et al. 2004), *D. ezoana*, *D. kanekoi*, and *D. littoralis* are included in the *montana* phylad, but our tree has the deepest branch separating these species, along with the *virilis* phylad, from the *montana* phylad. We suggest that the clearest resolution to this is to propose three phylads within the group: the *montana* phylad, containing *D. montana*, *D. lacicola*, *D. flavomontana*, and *D. borealis*; the *virilis* phylad, containing *D. virilis*, *D. lummei*, *D. novamexicana*, and *D. americana* (and *D. texana*, not sampled here); and a *littoralis* phylad, containing *D. littoralis*, *D. ezoana*, and *D. kanekoi* (Fig. 1a). Within the *virilis* phylad, there is maximal support for divergence of *D. virilis* and *D. lummei* before the Nearctic *americana* clade, consistent with previous descriptions (Nurminsky et al. 1996; Caletka and McAllister 2004; Orsini et al. 2004; Wang et al. 2006; Chekunova et al. 2008; Morales‐Hojas et al. 2011). Because no whole‐genome data for *D. texana* are available, we could not fully resolve relationships within the *americana* clade. In comparison to the *virilis* phylad, there has been some disagreement regarding the placement of species within the *montana* phylad. In contrast to previous work, we show that species within the *montana* phylad are monophyletic with two sister lineages including the species pairs *D. borealis* and *D. flavomontana*, and *D. montana* and *D. lacicola*.

We dated species divergence with an independent analysis using putatively neutral short introns (<80 bp) randomly sampled genome wide, using a previously adopted calibration point at the node characterizing the split of the *americana* clade from *D. lummei* around 2.7–3.1 MYA, at the onset of the Northern Hemisphere Glaciation (Caletka and McAllister 2004; Morales‐Hojas et al. 2011). Our inferred date for the basal node of the *virilis* group is 9 MYA, with the *littoralis* and *virilis* phylads diverging later on around 7.5 MYA (Fig. 1a). Interestingly, the *littoralis* phylad radiated earliest and therefore contains the oldest species within the *virilis* group, with radiation of the *virilis* and *montana* phylads occurring 3.8 and 4.7 MYA, respectively. Consistent with previous analyses, we show that divergence of the *americana* group within North America occurred relatively recently (Caletka and McAllister 2004; Morales‐Hojas et al. 2011). To examine these divergence time estimates further, we also scaled parameter estimates using the *Drosophila melanogaster* mutation rate (2.8 × 10^−9^) and a generation time of 0.75 to incorporate variation in voltinism across the group. These estimates were largely consistent with those scaled by the calibration point (Table S2), but with slightly earlier divergence times across all nodes.

Phylogenetic relationships and the existence of three distinct clusters were reflected across both the autosomes and the X chromosome in principal component analysis (PCA) (Fig. 1b). Additionally, PCA showed that species within the *montana* phylad show tight clustering despite lineages diverging ∼4 MYA. Finally, we found no difference between mean divergence times estimated from coding regions in autosomes and the X chromosome (*W* = 42, *P*‐value = 0.5) (Table S3).

### INTROGRESSION IS PERVASIVE BETWEEN SPECIES OF THE *virilis* GROUP

Although we recovered phylogenetic relationships with complete bootstrap support across the group, we note that relying solely on bootstraps to determine uncertainty in phylogenetic relationships may be misleading because maximal bootstrap support may often coincide with model misspecification (Yang and Zhu 2018), or considerable underlying gene tree conflict and systematic error (Kumar et al. 2012; Salichos and Rokas 2013). Using IQTREE2, we examined levels of gene and site discordance. For every branch in the species tree, gene and site concordance factors are defined as the proportion of gene trees and sites in a given loci that are in agreement with the species tree. Branches leading to the *D. kanekoi* and *D. ezoana* species pair and the branch leading to *D. littoralis* showed only 18% and 26% of decisive gene trees supported each respective branch in the species tree. Similarly, the same branches showed high levels of site‐level phylogenetic discordance with around half the number of decisive sites (44% and 47%, respectively) found to be concordant with the species tree (Figs. S2, S3). Additionally, the branch leading to the *D. borealis* and *D. flavomontana* species pair showed considerable levels of gene‐ (29%) and site‐level discordance (63%). In all three cases, the placement of these branches has been the most difficult to resolve in previous phylogenies (Morales‐Hojas et al. 2011). The *virilis* phylad, on the other hand, had comparatively lower levels of gene tree discordance (Fig. S2).

**Figure 2:**
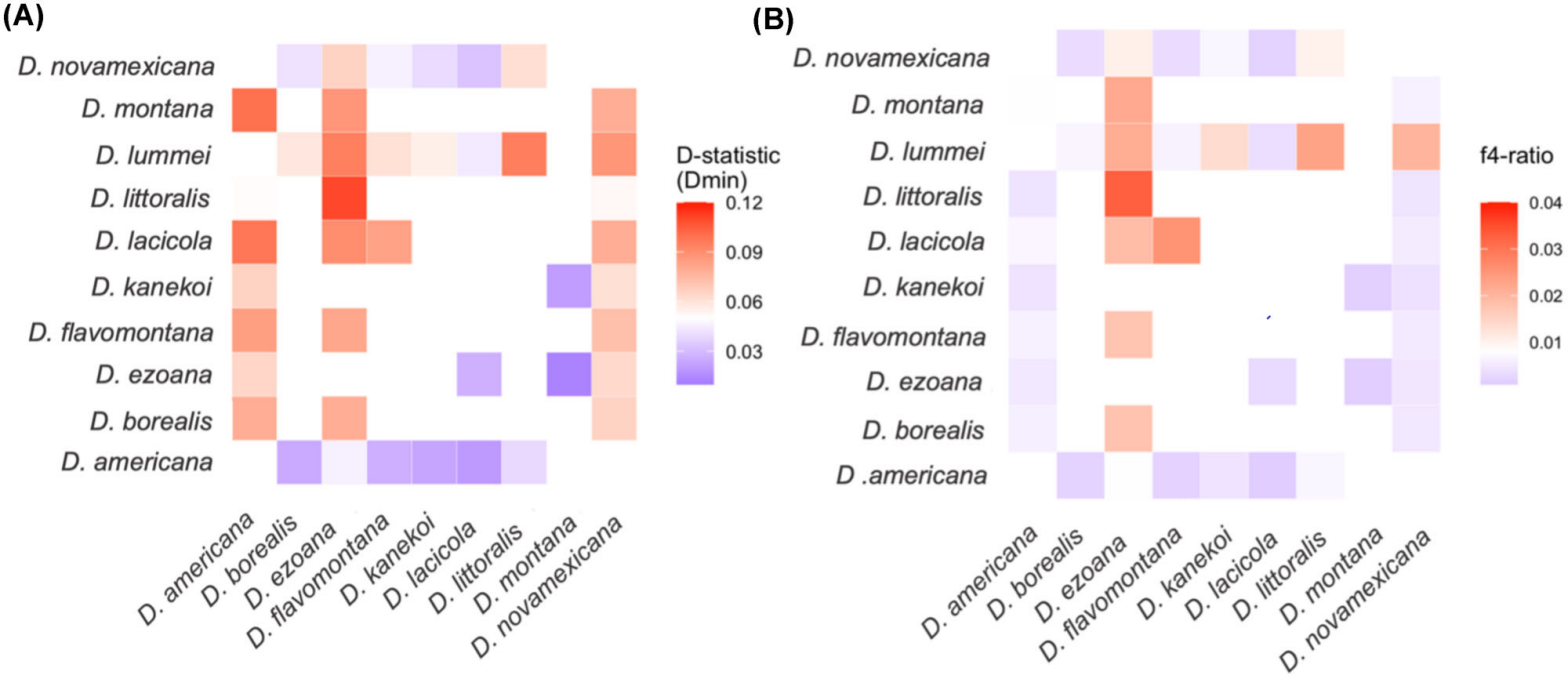
Gene flow is extensive between Palearctic and Nearctic members of the *montana* and *littoralis* phylads. (a) *D*‐statistic values shown between species with significant, excess allele sharing. (b) Admixture proportions (f4‐ratio) between species with evidence of significant, excess allele sharing

Although underlying gene tree conflict may be the consequence of poor phylogenetic signal or other technical issues associated with tree inference, it may also reflect genuine signals of gene flow or incomplete lineage sorting (ILS). To determine whether gene flow has occurred, we tested for excess shared, derived variants by calculating the minimum *D*‐statistic (*D*_min_) for each trio in the group, where trios were conservatively organized to minimize the amount of introgression detected (Malinsky et al. 2020). Thirty‐five of the 120 trios tested (35%) showed significant excess shared alleles after correcting for multiple testing, with mean excess allele sharing of 0.06 (6%). These results are incompatible with a single tree relating all species within the group and ILS, and provide strong evidence for gene flow between some species. Additionally, we calculated f4‐ratios, which estimate the amount of ancestry in an admixed population that comes from potential donor populations. We found only 12 trios show significant excess allele sharing (*D*_min_) and ancestry proportions (f4‐ratio) over 1% (Fig. 2). Introgression between *D. littoralis* and *D. ezoana* was consistently supported. We also found support for introgression between *D. lummei*, and *D. littoralis* and *D. ezoana*. Finally, we found consistent signals of introgression between *D. ezoana*, distributed in the northern parts of Europe, Asia, the Far East, and in Japan, and the North American species of the *montana* and *virilis* phylad, which indicates that the ancestors of these species must have had overlapping distributions.

To investigate potential ancient introgression events, we calculated *D*‐statistics by organizing trios according to their species tree relationships, maximizing the potential to detect gene flow events, as opposed to minimizing the *D*‐statistic (*D*_min_) to calculate conservative estimates of introgression. Only 13 trios (out of 120 tested) showed significant *D*_min_ values exceeding 0.1 after correcting for multiple testing. These were mostly between species within the *montana* and *littoralis* phylads. To distinguish between individual gene flow events between multiple species, or a single ancestral gene‐flow event affecting multiple descendant lineages, we calculated the f‐branch metric (ƒ_b_(C)) (Malinsky et al. 2018). We found evidence for ancestral gene flow, with the branch leading to the *littoralis* phylad showing the highest level of introgression with *D. montana* (*f*_b_(C) = 28%; *P* < 0.001; Fig. S4), and similarly high levels of introgression between the ancestral branch of the *littoralis* phylad and the other species of the *montana* phylad. This indicates that an ancestral gene flow event is at least partially responsible for the allele sharing between these species.

We tested for local phylogenetic discordance within phylads using TWISST. Such discordance can arise either due to gene flow or ILS. The species tree was the most well‐represented topology within each phylad. However, within the *montana* phylad, discordant topologies often showed comparable weighting to that concordant with the species tree across chromosomes (Fig. S5a). This may suggest a lack of phylogenetic signal, consistent with ILS genome‐wide as well as heterogeneous patterns of gene flow. On the X chromosome, we found a discordant topology with *D. borealis* and *D. lacicola* as this species pair had a higher average topology weighting than the species tree topology (ANOVA: df = 2, *F* = 1408.4, *P* < 0.001; Tukey multiple comparisons test: *P* < 0.001), suggesting extensive introgression on this chromosome. The other trios showed more consistent patterns of gene concordance across all chromosomes. Hence, it seems that interspecific gene flow (or ILS) may be more prominent in the *montana* phylad than in the *littoralis* and *virilis* phylads.

### LOCALIZED PATTERNS OF GENOME‐WIDE INTROGRESSION

**Figure 3:**
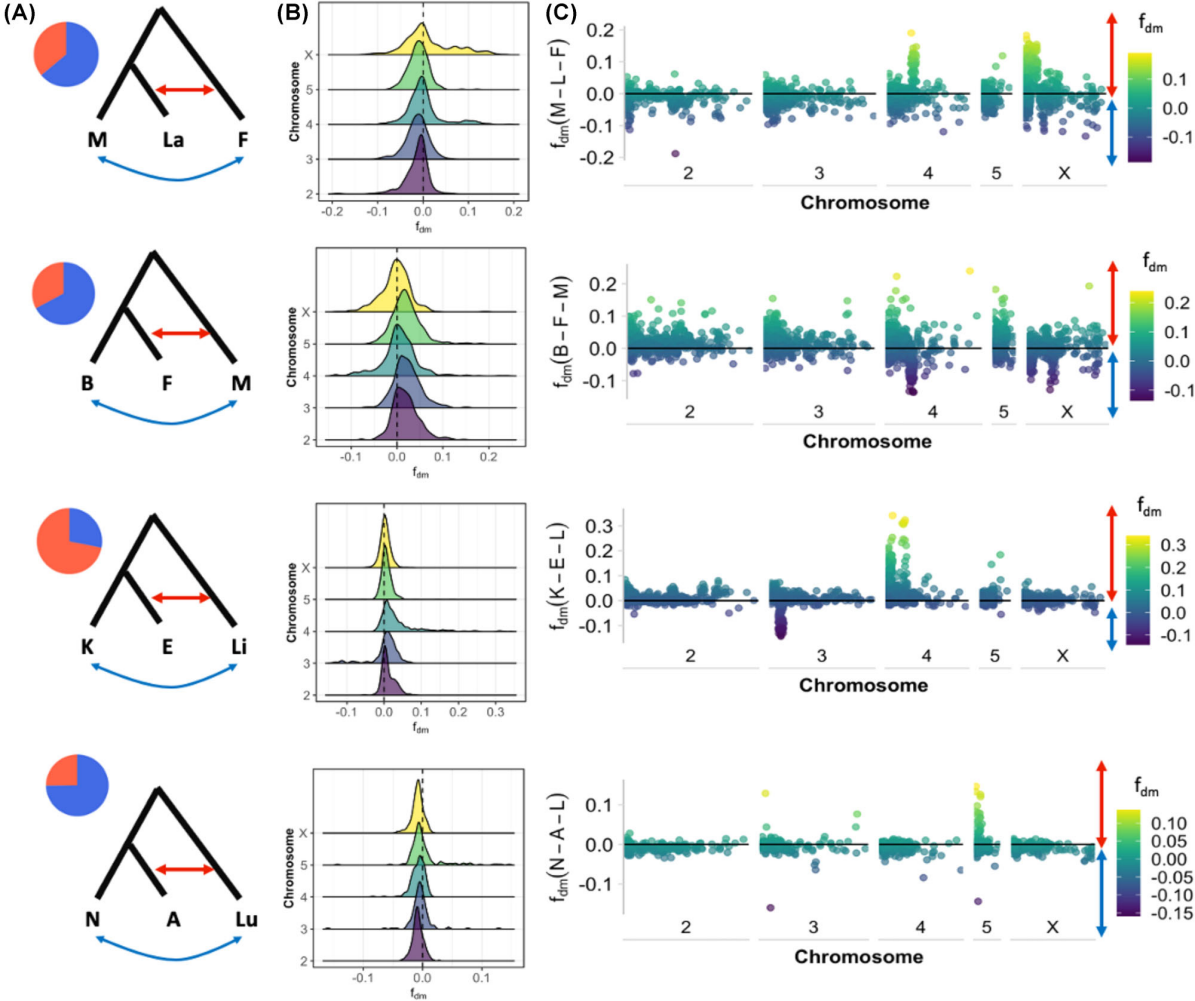
Genome‐wide introgression facilitates phylad‐specific gene sharing. (a) Schematic showing directionality of gene flow tested by *f*_dm_ statistic and pie chart illustrating proportion of introgression between species in each comparison calculated using *f*_dm_. (b) Distributions of introgression across chromosomes for each comparison. Dotted line indicates neutrality (no allele sharing). (c) Genome‐wide introgression shown across chromosomes for each comparison, with solid line indicating neutrality. Here, species names are abbreviated (M: *D. montana*, La: *D. lacicola*, B: *D. borealis*, F: *D. flavomontana*, E: *D. ezoana*, Li: *D. littoralis*, K: *D. kanekoi*, N: *D. novamexicana*, Lu: *D. lummei*; and A: *D. americana*. Asterisks on topologies represent topologies that are concordant with the species tree.

Additionally, we calculated genome‐wide admixture proportions (*f*_dm_ and *f*_d_) in sliding windows. We found higher mean admixture proportions between species within the *montana* phylad (La ← F mean *f*_d_ = 0.04441; F ← M mean *f*_d_ = 0.04659; E ← Li mean *f*_d_ = 0.04065) compared to the *virilis* phylad (A ← Lu mean *f*_d_ = 0.02007), indicating evidence of recent admixture between sympatric species. The directionality of *f*_dm_ suggests considerable gene flow has occurred between *D. montana* and both *D. borealis* and *D. flavomontana* (Fig. 3). In the *littoralis* phylad, we found that admixture has mostly occurred between *D. ezoana* and *D. littoralis*, with peaks of admixture localized to chromosome 4. Contrary to expectations, we found evidence of significantly higher admixture on the X chromosome between *D. lacicola* and *D. flavomontana*, when compared to autosomes (ANOVA: df = 4, *F* = 48.943, *P* < 0.001; Tukey multiple comparisons test: *P* < 0.001). This is not the case for other comparisons across the *virilis* phylad, where admixture proportions are usually considerably lower on the X chromosome compared to the autosomes (F ← M ANOVA: df = 4, *F* = 6.5482, *P* < 0.001; E ← Li ANOVA: df = 4, *F* = 64.584, *P* < 0.001) (Table S6).

To look for instances of overlap in admixture between the four trios tested, we extracted the admixture outlier windows (*f*_d_, 95% quartile) for all trios and assessed overlap between these. We found little overlap between windows showing admixture proportions, with only one showing overlap between two trios. We performed gene ontology analysis for biological processes on genes found within the top 20 windows with the highest (10) and lowest (10) admixture proportions (*f*_dm_) for all trios. Introgressed genes between *D. montana*, *D. borealis*, and *D. flavomontana* showed significant (*P* = 4.98 × 10^–14^) enrichment for heat shock proteins involved in polytene chromosome puffing and in insect stress responses (Zhao and Jones 2012) (Fig. S6; Table S7).

### PATTERNS OF GENOME‐WIDE DIVERGENCE, GENE FLOW, AND REPRODUCTIVE ISOLATION

**Figure 4:**
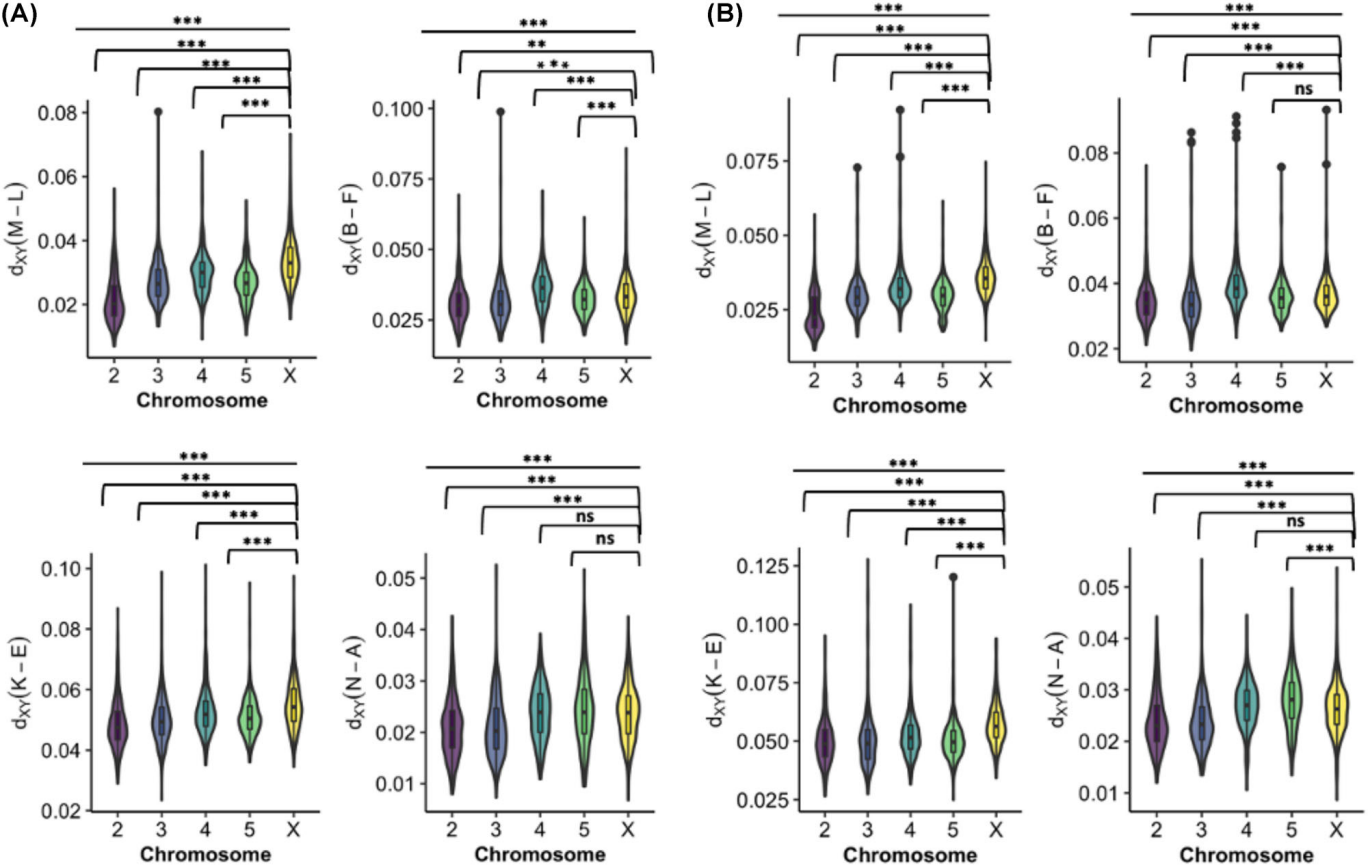
Absolute divergence calculated for each chromosome across coding and noncoding regions. *d*_XY_ was calculated using genome‐wide ∼50‐kb windows between species pairs in the *virilis* group. (a) *d*_XY_ was calculated for coding regions across the four species pairs and (b) for intergenic regions. The species pairs shown here are the following: *D. montana* and *D. lacicola* (M–L), *D. borealis* and *D. flavomontana* (B–F), *D. kanekoi* and *D. ezoana* (K–E), and *D. novamexicana* and *D. americana* (N–A). Significance between all chromosomes was tested using an ANOVA and significance between the X chromosome and each of the autosomes was tested using the Tukey test. Stars indicate level of significance: 0 = ***, 0.001 = **, 0.01 = *, 0.05 = ., and >0.05 = ns.

We calculated absolute divergence in coding and noncoding regions between species pairs to identify any differences between chromosomes, and to detect possible “faster X” divergence. We found significant differences in absolute divergence between all chromosomes in noncoding and coding regions. However, inconsistent with expectations, we found only limited evidence for any faster X effect in species pairs across the *virilis* group in both coding and noncoding regions. Specifically, only *D. montana* and *D. lacicola* (TMRCA: ∼2 MYA), and *D. kanekoi* and *D. ezoana* (TMRCA: ∼6 MYA) showed significant, elevated divergence on the X chromosome relative to the autosomes (M–L ANOVA: df = 4, *F* = 218.41, *P* < 0.001; K–E ANOVA: df = 4, *F* = 59.086, *P* < 0.001) (Fig. 4). Between *D. borealis* and *D. flavomontana*, and *D. novamexicana* and *D. americana*, we found the X chromosome (B–F mean X genic *d*_XY_: 0.034; N–A mean X chromosome genic *d*_XY_: 0.023) to show lower divergence compared to chromosome 4 (B–F mean chromosome 4 genic *d*_XY_: 0.036) and chromosome 5 (N–A mean chromosome 5 genic *d*_XY_: 0.024), respectively.

To explore this further, we characterized migration rates for autosomes and the X chromosome separately. We sampled short (200–300 bp) intergenic blocks for three species per comparison including a single sequence per species pair, and an outgroup sequence (*D. virilis*). We sampled 9949 short intergenic blocks across the autosomes and 11,127 across the X chromosome. These were analyzed using 3s, a coalescent‐based maximum likelihood tool estimating divergence, effective population size, and migration parameters under a strict divergence model and an isolation with migration model (Dalquen et al. 2017).

Using the isolation with migration model, we found different levels of postdivergence migration between the autosomes and the X chromosome, and also differences in the directionality of gene flow within pairs. Between *D. montana* and *D. lacicola*, we observed higher levels of migration from *D. montana* into *D. lacicola* (0.045–0.064 migrants per generation) than from *D. lacicola* into *D. montana* (0.026–0.015 migrants per generation) on both autosomes and the X chromosome. This difference between *D. montana* and *D. lacicola* is larger on the X chromosome, suggesting potential stronger X chromosome barriers in *D. montana* compared to *D. lacicola*. We found similar bidirectional gene flow between *D. borealis* and *D. flavomontana* on the autosomes (0.076 and 0.08 migrants per generation), but higher levels of gene flow from D*. flavomontana* into *D. borealis* (0.095 migrants per generation) than from *D. borealis* into *D. flavomontana* (0.039 migrants per generation), on the X chromosome. However, in both these cases, the gene flow model did not fit significantly better than a strict divergence model (Table S8).

Between *D. kanekoi* and *D. ezoana*, we found an order of magnitude difference in levels of gene flow between the autosomes (0.046 and 0.059 migrants per generation) and X chromosome (0.353 and 0.351 migrants per generation), with considerably higher levels of bidirectional gene flow found on the X chromosome, suggesting potential maintenance of introgressed genetic variation on the X chromosome relative to the autosomes. Finally, we observed evidence of unidirectional gene flow between *D. americana* and *D. novamexicana* on both the autosomes and the X chromosome, suggesting a potential role for reciprocal autosomal and X chromosomal barriers. However, once more the gene flow model did not fit significantly better than the strict divergence model for both pairwise comparisons.

To understand broad patterns of reproductive isolation within the group, we collected data on premating isolation and biogeography from Throckmorton (1982) and Yukilevich (2014), and paired this with genome‐wide estimates of divergence (*d*_XY_) across the group. Specifically, previous comparative surveys of *Drosophila* have shown that prezygotic barriers generally evolve faster in sympatric species pairs compared to allopatric species pairs (Coyne and Orr 1997, 1989). After transforming estimates of *d*_XY_, premating isolation, and biogeography into dissimilarity matrices, we asked whether there was an association with biogeography—whether pairs share overlapping ranges (i.e., sympatry) or not (i.e., allopatry)—and estimates of premating isolation. We found a significant positive correlation between biogeography and premating isolation, while controlling for the effect of genome‐wide divergence (Partial mantel test; *R* = 0.1823, *P* = 0.016, permutations = 10,000; Fig. S7; Table S9). Sympatric pairs showed higher premating isolation than allopatric pairs. Notably, three species comparisons (*D. montana*, *D. borealis*, and *D. lacicola*) showed high levels of premating isolation and low genome‐wide *d*_XY_ compared to most other species comparisons, indicating rapid evolution of premating barriers.

## Discussion

A major challenge in speciation genomics is the identification of genetic variation associated with the emergence and maintenance of reproductive barriers. One way of addressing this is by characterizing genetic divergence, gene flow, and the strength of reproductive barriers in species pairs across the speciation continuum. For example, a comparative survey examining rates of migration and genomic divergence across animals found “a gray zone of speciation,” where at 0.5%–2% net divergence a cessation of gene flow was observed (Roux et al. 2016). Collectively, these analyses have yielded important, general observations: (a) genome‐wide effective migration rate reduces with higher levels of genetic divergence; (b) heterogenous patterns of genome‐wide genetic divergence are expected to be the combined result of gene flow, divergent selection, and genomic features such as recombination rate; (c) sex chromosomes show higher genetic divergence compared to autosomes; and (d) sexual isolation is generally higher in sympatric species pairs compared to allopatric species pairs (Coyne and Orr 1997, 1989). In *Drosophila*, broad‐scale comparative analyses have so far been limited to single species pairs or complexes, with only few exceptions (Mai et al. 2020; Suvorov et al. 2022; ). Using de novo whole‐genome data and genome assemblies, we examined the prevalence of phylogenetic discordance, gene flow, levels of genome‐wide divergence, and measures of reproductive isolation in the *D. virilis* group.

Phylogenetic placements in the *virilis* group have been the source of some contention. Previous inferred phylogenies have suggested that *D. littoralis*, *D. kanekoi*, and *D. ezoana* are more closely related to the *montana* phylad than to the *virilis* phylad (Orsini et al. 2004; Andrianov et al. 2010). In agreement with Morales‐Hojas et al. (2011), we found that these species are more closely related to the *virilis* phylad, but are quite distinct, and we suggest that they represent an additional phylad within the group, termed *littoralis* after the oldest species. We also resolved species pairs within the *montana* phylad. Importantly, we show that the difficulty in resolving the phylogeny has likely been the result of pervasive gene flow between closely related species within the same phylads, and ancient introgression between the common ancestors of the phylads. Using a combination of *D*‐statistics and gene‐ and site‐concordance factors, we detected evidence of strong phylogenetic incongruence in branches leading to the *littoralis* phylad. Between closely related species, we also found considerable heterogeneity in genome‐wide phylogenetic incongruence likely attributable to more recent gene flow between species, particularly in the *montana* phylad where species often share overlapping geographic ranges (Fig. 1c).

We quantified divergence times for species in the group using putatively selectively neutral small intronic regions (Haddrill et al. 2005; Halligan and Keightley 2006) and a single calibration point used previously for this group. The oldest radiation within the group is that of the *littoralis* phylad, which is ∼7.5 MYO. Little is known about the ecology and distribution of *D. kanekoi*, except that its range is limited and largely endemic to Japan, alongside more widespread species in the group like *D. montana*, *D. virilis*, and *D. ezoana*. *Drosophila littoralis* and *D. ezoana* are found in northern Finland together with *D. montana* and *D. lummei* (Aspi et al. 1993). The divergence of the *montana* and *virilis* phylads occurred later, around the beginning of the Pliocene, where temperatures likely warmed and passage into North America via the Bering Strait was possible (Marincovich and Gladenkov 1999; Robinson et al. 2008). Divergence of both *D. montana* and *D. lacicola*, and *D. borealis* and *D. flavomontana* in the *montana* phylad roughly coincide with the split of *D. lummei* from the *americana* clade in the virilis phylad and the intensification of the Northern Hemisphere Glaciation (Caletka and McAllister 2004; Bartoli et al. 2005; Ruggieri et al. 2009). Altogether, we suggest that changes in climate during the Pliocene have facilitated the spread and early divergence of both the *virilis* and *montana* phylads.

The phylogeographic origin of the *montana* phylad has been the subject of recent debate. Unlike other members of the *montana* phylad, whose ranges are restricted to North America, *D. montana* coexists in parapatry with *D. flavomontana*, *D. borealis*, and *D. lacicola* in North America, and overlaps with *D. ezoana* and *D. littoralis* in Eurasia and with *D. ezoana* and *D. kanekoi* in Japan (Fig. 1). Our results indicate that the divergence of *D. montana* and *D. lacicola* occurred ∼2 MYA, although little is known about the range and ecology of *D. lacicola*, therefore it is difficult to determine what may have driven this. Recent modeling of divergence time between *D. montana* and *D. flavomontana* (Poikela et al., in prep) suggested similar divergence time estimates (3–4 MYA). In both this study and previous attempts to scale divergence time estimates (Caletka and McAllister 2004; Morales‐Hojas et al. 2011), we assumed that the ancestor of *D. lummei* and the *americana* clade had a Holarctic distribution, and with precipitous temperature decline around 3 MYA, gene flow between *D. lummei* and the ancestor of the *americana* clade ceased. We also scaled divergence time estimates using a *D. melanogaster* point mutation rate (2.8 × 10^–9^) (Keightley et al. 2014). Here, divergence time scaling is not biased by a priori expectations. We found these divergence time estimates to be largely consistent (see Table S2), with mutation rate estimates indicating marginally earlier divergence times. Biogeographical scenarios associated with the split of the *montana* phylad are varied. For example, hypothetical earlier estimates may be consistent with divergence of *D. montana* and the *montana* phylad in North America, whereas older estimates may indicate a potential origin for *D. montana* in Asia (Mirol et al. 2007; Morales‐Hojas et al. 2011; Garlovsky et al. 2020). However, given the range of *D. montana*, it is possible that the ancestral lineage of the *montana* phylad had a Holarctic distribution leading to vicariant speciation events in North America.

Interspecific hybridization is common across closely related species, but the question of when gene flow is expected to cease between distantly related species is unclear. We found only 10% of tested trios to show any evidence of gene flow. However, after accounting for phylogeny and nonindependence of gene flow estimates using the f‐branch statistic (Malinsky et al. 2020), we only found strong evidence for an ancient gene flow event(s) between the ancestor of *montana* phylad and the *littoralis* phylad lineage, prior to the divergence of *montana* phylad. We found no evidence for independent gene flow events between *D. montana* and *D. littoralis*, *D. ezoana*, and *D. kanekoi*, suggesting either that strong barriers to gene flow arose quickly in Eurasian populations of *D. montana* or that the lack of gene flow is indicative of divergence of the *montana* phylad in North America. These analyses demonstrate the difficulty in disentangling recent hybridization events from ancient hybridization events across relatively speciose taxonomic groups (Malinsky et al. 2018; Ferreira et al. 2020).

Interpreting patterns of genetic divergence is difficult due to the effect of recombination rate and linked selection (Noor and Bennett 2009; Cruickshank and Hahn 2014). Although absolute divergence is not affected by within‐population diversity and linked selection, it is expected to be affected by recombination rate variation and gene flow, such that divergence is lower in regions of low recombination and gene flow can homogenize peaks of divergence (Nachman and Payseur 2012). Here, we observed heterogenous patterns of divergence across “true” species pairs, and in striking contrast with previous analyses across diverse taxonomic groups (Meisel and Connallon 2013; Charlesworth et al. 2018), we found unexpected patterns of low genetic divergence on the X chromosome compared to autosomes in two of the four *species pairs*. In the *montana* phylad, we observed evidence for a faster X chromosome effect between *D. montana* and *D. lacicola*, but not between *D. borealis* and *D. flavomontana*. One potential explanation for these patterns is differences in rates, direction, and timing of gene flow between species in the *montana* phylad. Our local introgression analysis (*f*_dm_) suggests most introgression has occurred recently between *D. montana*, *D. borealis*, and *D. flavomontana*, with some evidence of comparable levels of shared variation on the X chromosome and autosomes. But there has been extensive introgression between the X chromosomes of *D. borealis* and *D. lacicola*. Additionally, our data do not allow us to characterize or describe inversions despite their prevalence in the *virilis* group (Reis et al. 2018, 2020).

One way to understand differences in rates of gene flow between autosomes and the X chromosome is to explicitly model gene flow on autosomes and X chromosomes separately using isolation with migration (IM) models. Consistent with the expectations from our local introgression analyses (*f*_dm_), we found stronger unidirectional gene flow from *D. montana* into *D. lacicola*, and stronger gene flow from *D. flavomontana* into *D. borealis*. In both cases, the inferred rate of migration was stronger on the X chromosome compared to the autosomes. Counter to expectation, in both local introgression analyses and IM modeling, gene flow is greater between X chromosomes than autosomes. Most genomic characterizations of genome‐wide introgression in *Drosophila* (Meisel and Connallon 2013; Charlesworth et al. 2018) have found overwhelming support for less introgression on the X chromosome (Turissini and Matute 2017; Meiklejohn et al. 2018; Mai et al. 2020), and similar patterns have been observed in *Heliconius* (Van Belleghem et al. 2018), humans (Sankararaman et al. 2014), mice (Payseur et al. 2004), and other groups (Presgraves 2018). In *D. kanekoi* and *D. ezoana*, we find considerably higher estimates of migration rates on the X chromosome relative to autosomes, suggesting barriers to gene flow are more often autosomal. The addition of polymorphism data would likely increase chances to reliably detect rates of unidirectional migration across the group (Dalquen et al. 2017).

The role of introgression on adaptation and speciation has received considerable attention. In some cases, the transfer of beneficial alleles into recipient taxa can facilitate adaptation (Whitney et al. 2010, 2006; Dasmahapatra et al. 2012; Racimo et al. 2016; Malinsky et al. 2018; Oziolor et al. 2019; Valencia‐Montoya et al. 2020). The exchange of locally adaptive introgressed variation can also contribute to speciation, whereas globally adaptive introgressed variation cannot (Abbott et al. 2013). However, reliably identifying adaptive introgression remains challenging. We found heterogenous patterns of introgression, with peaks specific to each trio. Additionally, functions of genes found within high‐confidence introgressed windows were trio specific, even between closely related species in the *montana* phylad, suggesting independent gene flow events and/or selective purging of introgressed variation following introgression events.

Reproductive barriers between species in the *virilis* group have been well‐characterized and contribute to comparative surveys of reproductive isolation and genetic divergence in *Drosophila* (Throckmorton 1982; Coyne and Orr 1997, 1989; Yukilevich 2014). Here, we recapitulate some of the patterns by demonstrating an association between biogeography and premating isolation, where sympatric species comparisons show higher levels of premating isolation compared to allopatric species. In particular, most species in the *montana* phylad show strong premating isolation despite relatively low genome‐wide divergence (and evidence of gene flow), indicating a complex demographic history and possible reinforcement of existing premating barriers. This is supported by recent work showing almost complete reproductive isolation between *D. montana* females and *D. flavomontana* males with evidence of reinforcement in the reciprocal cross in sympatric populations, as well as cascade reinforcement of these barriers between *D. flavomontana* populations (Poikela et al. 2019).

## Conclusion

The *virilis* group diverged relatively rapidly and evolved varied, but strong, isolation mechanisms in the face of gene flow. Ancient gene flow between the *montana* and *littoralis* phylads likely confused previous attempts to fully reconstruct the speciation history of the group. Within the *montana* phylad, introgression has been extensive, likely reflecting a recent history of gene flow between closely related species. We suggest that genes evolving rapidly throughout the group may play a role in sexual isolation and that introgressed variation has played a role in early divergence. We find evidence that gene flow may have contributed to reinforcement within the *montana* phylad. Differences in genetic divergence across chromosomes do not clearly support a disproportionate role for sex chromosome in facilitating isolation between species pairs in the *virilis* group. Our study clarifies the phylogenetic relationships between species in the *virilis* group and highlights the potential for gene flow to shape evolutionary history and patterns of genetic variation.

## AUTHOR CONTRIBUTIONS

MGR, VT, and AH conceived the study. MGR and VT designed the study. VT performed fieldwork and lab work. LY analyzed the data, with contributions from VT (genome assembly). LY wrote the manuscript with input from MGR, AH, and VT.

## CONFLICT OF INTEREST

The authors declare no conflict of interest.

## DATA ARCHIVING

Whole‐genome sequencing reads are available at the NCBI Sequence Read Archive under BioProject PRJNA855919. Genome assemblies and representative genome annotations can be found here: https://zenodo.org/record/7097273#.Yy3AVSHMLil. Scripts for analysis are available at https://github.com/LeebanY/Virilis_Comparative_genomics.

## Supporting information

Supplement Material

Supplement Material

Supplement Material

Supplement Material

Supplement Material

Supplement Material

Supplement Material

Supplement Material

Supplement Material

Supplementary figure 1: Detection of genes under putative positive selection in the virili*s* group
Supplementary figure 2: Gene concordance across nodes and chromosomes on the species tree
Supplementary figure 3: Site concordance across nodes and chromosomes on the species tree
Supplementary figure 4: Summary of f‐branch (f_b_) tests for introgression in the *virilis* group
Supplementary figure 5: Topology weighting shows widespread phylogenetic discordance across the virilis group
Supplementary figure 6: Gene ontology for genes showing signatures of admixture between trios.
Supplementary figure 7: Absolute genetic divergence against pre‐mating isolation for species pairs across the *virilis* group
Supplementary figure 8: Mean admixture proportions for every scaffold was plotted for closely‐related trios across the *virilis* group, against absolute genetic divergence in ‘true’ species pairs
