# Supplementary material for: Divergence and introgression among the virilis group of Drosophila
Source: Evol Lett. 2022 Nov 28;6(6):537–51. doi: 10.1002/evl3.301 (PMC9783487; doi:10.1002/evl3.301)
Supplement: Supplementary file 10 — Supplementary figure 1: Detection of genes under putative positive selection in the virilis group Supplementary figure 2: Gene concordance across nodes and chromosomes on the species tree Supplementary figure 3: Site concordance across nodes and chromosomes on the species tree Supplementary figure 4: Summary of f‐branch (fb) tests for introgression in the virilis group Supplementary figure 5: Topology weighting shows widespread phylogenetic discordance across the virilis group Supplementary figure 6: Gene ontology for genes showing signatures of admixture between trios. Supplementary figure 7: Absolute genetic divergence against pre‐mating isolation for species pairs across the virilis group Supplementary figure 8: Mean admixture proportions for every scaffold was plotted for closely‐related trios across the virilis group, against absolute genetic divergence in ‘true’ species pairs [file EVL3-6-537-s008.docx]

# Supplementary Methods

## Stocks; obtaining and sequencing samples.

For genome sequencing we used individual flies (female or male) which were derived from isofemale strains collected at different locations or received from the stock centers (for more details see Supplementary table 1). DNA was extracted with the CTAB method followed by RNAse treatment and multiple phenol-chlorophorm-isoamyl alcohol and chlorophorm-isoamyl alcohol cleaning steps. DNA integrity was assessed with agarose gel and the quantity was measured with qubit (Thermo Fischer Scientific).

## Genome assembly and annotation

The samples were prepared using Nextera libraries protocol and sequenced on Illumina HiSeq 2500 platform (150bp paired-end reads) at Edinburgh Genomics, UK. The read quality was checked with fastqc (Andrews 2010) and the assemblies were done with MaSuRCa genome assembler (Zimin et al. 2013). Genome quality was assessed using BUSCO (v.2.0) with the Diptera gene set (Simão et al. 2015). To annotate the genomes we used BRAKER (v.2.1.4 ) with GenomeThreader to map D. *virilis* homologs, which was used as evidence, and subsequently used to train ab initio gene prediction via AUGUSTUS (Stanke et al. 2006; Hoff et al. 2016). We then filtered predicted genes using AGAT program ‘agat_sp_filter_incomplete_gene_coding_models.pl’ (https://github.com/NBISweden/AGAT) for genes without a start and stop codon.

## Read mapping, variant calling and filtering

FASTQ reads were first trimmed using Trimmomatic (v0.36) with the following parameters (ILLUMINACLIP:TruSeq3-SE:2:30:10LEADING:3TRAILING:3SLIDINGWINDOW:4:15 MINLEN:36) (Bolger et al. 2014). Reads for all samples, including the D. *virilis* and *D. americana* genomes obtained from Flybase, were mapped to a PacBio reference genome (Poikkela et al, in prep) using bwa-mem (0.7.15)(Li 2013). We used samtools (v.1.6) to sort, mark and remove duplicate reads, and subsequently, we used to GATK Genome Analysis tool to realign reads around indels. We called variant and invariant sites using bcftools (v.1.6) and filtered sites using bcftools for depth (DP>10) and mapping quality (MQ>30) (Danecek et al. 2021).

## Resolving the phylogenetic tree and divergence times

To investigate phylogenetic relationships within the *virilis* group, we utilised proteins characterised as complete and single-copy for each species using BUSCO. For species with multiple de-novo genomes, we selected protein sets with the highest BUSCO completeness percentage. After retrieving BUSCO protein sets for each species, we used Orthofinder (v2.3.12) to identify orthologs from species-specific protein sets (Emms and Kelly 2015, 2019). Multiple sequence alignments of single-copy orthologs were retrieved from Orthofinder and subsequently aligned using MAFFT (v7.147b). We then filtered gappy and poorly-aligned columns from all alignments using trimAl (v1.4.rev15) with -gappyout option. In total, 1336 alignments remained and were used for phylogenetic analysis. We inferred a maximum likelihood phylogeny using a concatenated alignment of these single-copy orthologs and a JTT+F+R5 model determined by ModelFinder (Kalyaanamoorthy et al. 2017) and 1000 bootstraps in IQTREE (v.2.0.3) (Nguyen et al. 2015). Concatenation of alignments produced 787, 425 bp sequences for all 12 species, with 18,827 parsimony-informative sites and 64,533 singleton sites. Since concatenating loci can result in failure to resolve the species tree (Mendes and Hahn 2018), we also inferred individual gene trees using maximum likelihood in IQTREE2 for each gene alignment and subsequently used these for species tree reconstruction in ASTRAL (v5.6.3)(Mirarab et al. 2014). Given a set of gene trees, ASTRAL constructs a species tree from the maximum number of quartet trees represented in all given gene trees, thereby accounting for possible gene tree discordance. In both the concatenated maximum-likelihood phylogeny and species tree, *D. mojavensis* was used as the outgroup. Finally, to assess phylogenetic concordance amongst the gene trees, we estimated gene concordance and site concordance factors using IQTREE. Orthofinder was performed on BUSCO single-copy protein sequences for each genome to retrieve orthogroups.

To date divergence times and infer ancestral population size for each node, we first randomly extracted small introns (<80bp) from the VCF file using bedtools (v2.29.2) and VCFtools (0.1.14)(Quinlan and Hall 2010; Danecek et al. 2011). Here, small introns were chosen due to previous work in *Drosophila* showing selective constraint in small introns was comparable to selective constraint in selectively neutral, four-fold degenerate sites. Extracted small introns were then concatenated into small blocks of 300bp based on their proximity to one another. In total, we used 100 regions around 300bp long. We then converted these 300bp regions into multiple sequence alignments with the requirement that all species contain a SNP for each column in the alignment, using the script vcf2phylip.py. We estimated divergence time and ancestral population size under the multiple-species coalescent model using BPP (v.4.1.4) (Flouri et al. 2018). Specifically, we use the A00 model which estimates divergence time and population sizes using a fixed species tree and provided species-to-sample relationships. For this, we provided the species tree inferred by ASTRAL. We then converted divergence time estimates from coalescent units into real dates using a single calibration point at the node separating the Eurasian *D. lummei* from the American (*D. americana* and *D. novamexicana*) species. This calibration point has been used in previous analyses of virilis group phylogeny, and assumes the last common ancestor of *D. lummei* and the ‘*americana*’ clade had a Holarctic distribution and diverged following the Northern Hemisphere Glaciation event around 2.7-3.1 Mya (Caletka and McAllister 2004; Morales-Hojas et al. 2011). Additionally, we scaled divergence time estimates using the *Drosophila melanogaster* mutation rate 2.8 x 10^-9^ (Keightley et al. 2014), and a generation time of 0.75 to capture variation in voltinism for species in the group, where some species in the group are bivoltine in North American, southern populations and are univoltine in northern populations at higher altitudes (Moorhead 1954). For calibration and estimation of lower and upper-bound divergence, we used the bppr package in R.

Additionally, to identify differences in divergence and ancestral population size between autosomes and the X chromosome, we randomly extracted coding regions in the autosomes and X chromosomes separately, and produced 100 loci each consisting of 300bp coding sequences for autosomes and X chromosomes. Again, we used vcf2phylip.py to produce multiple sequence alignments containing columns where all samples contained SNPs – columns with missing data for any sample were discarded for each loci. We then estimated divergence times and ancestral population sizes for autosomes and the X chromosome using the A00 model in BPP.

Finally, we used principal component analysis to assess whether genome-wide variation supported species relationships found using orthologs. We randomly extracted coding regions across all genes in the variant call file (VCF) from the autosome and X chromosome separately and pruned SNPs using bcftools ‘+prune’ command with a window size of 100. We then used PLINK2 to further prune the dataset down using windows of 50kb, step sizes of 10 and r^2^ threshold of 0.1 (Purcell et al. 2007). For the X chromosome, 3,218 variants were used for the PCA, and for the autosomes 12,272 variants were used.

## Estimating gene flow

To test for introgression we calculated excess allele sharing using D-statistics (Green et al. 2010) using filtered variants (described above). Specifically, we calculated D_min_, defined as the minimum amount of allele sharing regardless of any assumptions made about the tree topology and species relationships, for each trio in the *virilis* group. Additionally, to more accurately determine D_min_, we provided the species tree constructed using ASTRAL as an input species tree. We corrected for multiple testing using a Bonferroni correction and *D* values with P < 0.05 were considered significant. Additionally, to determine when introgression may have taken place, we calculated the f-branch statistic (Malinsky et al. 2018, 2020). The f-branch statistic (ƒ_b_ (C)) measures admixture between a donor species C and branch *b* by calculating admixture for all possible combinations of ƒ(A,B,C;O), where A and B are sister lineages, and A is a descendant of branch *a* and B is a descendant of branch *b.* In short, The f-branch statistic (ƒ_b_ (C)) calculates the amount admixture that has occurred between taxon C and the branch (*b*) leading to a descendant taxon B, relative to the admixture that has occurred between taxon C and a sister branch to taxon B (branch *a*), where A is a descendant of branch *a* and B is a descendant of branch *b (*ƒ(A,B,C;Outgroup)). Significant f-branch (ƒ_b_) values indicate ancient introgression between branch *b* and taxon C. Admixture proportions were also calculated in windows using the f_d_ (Martin et al. 2015) and f_dm_ (Malinsky et al. 2015) statistic. Both statistics were calculated in windows of 200bp with a step size of 100bp.

To supplement this, we constructed genome-wide phylogenies and weighted topologies using TWISST (Martin and Belleghem 2017). Phylogenies were calculated in three clades: the *virilis* phylad, the *littoralis* phylad and the *montana* phylad. Phylogenies were constructed from filtered variants (described above) using the GTR model in PhyML in windows of 100bp. TWISST analyses were performed using the ‘complete’ parameter method, calculating exact topological weightings by considering all possible sub-trees. For all phylogenies, *D. virilis* was used as the outgroup.

Additionally, we converted the filtered VCF file into a genotype-specific file and then used popgenWindows.py to calculate genetic divergence (d_XY_) in 50kb windows for all possible species pairs (<https://github.com/simonhmartin/genomics_general>). To aid in Coyne & Orr-style (1989, 1997) comparative analysis, we retrieved data on pre-mating isolation and biogeography for species pairs in the group from (Yukilevich 2014), and where biogeography data was missing, we used biogeography inferences from Throckmorton (1982) to supplement the analysis.

To determine the degree of potential gene flow between all four species pairs following divergence, we used a maximum likelihood implementation of isolation-with-migration model in the ML 3s program (Dalquen et al. 2017). We randomly sampled intergenic blocks of 200-300bp of loci from autosomes and the X chromosome separately from the filtered VCF file using VCFtools (v0.1.14) and bedtools (v2.29.2). Blocks were then converted into phylip format using vcf2phylip.py script (https://github.com/edgardomortiz/vcf2phylip). We used a single representative whole-genome sequencing sample for all species in the four species pairs we considered. Here, we used *D.montana* (Fairbanks, Alaska, USA), *D.lacicola* (Manitoba, Canada), *D. borealis* (Lake Itasca State Park, Minnesota, USA), *D. flavomontana* (Crested Butte, Colorado, USA), *D. ezoana* (Oulanka, Finland*)*, *D. kanekoi (Hokkaido, Japan)*, *D. novamexicana* (Moab, Utah, USA) and *D. americana* (Mississippi, USA), with *D.virilis* used as the outgroup. The IM model uses three species ((A,B),C) where A and B are the in-group species for which unidirectional gene flow parameters are estimated (M_AB_ and M_BA_), and C is the outgroup involving no gene flow.

We tested for gene flow between four species pairs: 1) *D. montana* and *D. lacicola*, 2) *D. borealis* and *D. flavomontana*, 3) *D. ezoana* and *D. kanekoi*, and 4) *D. americana* and *D. novamexicana*, resulting in 4 comparisons for the autosomes and the X chromosomes, respectively (8 comparisons in total), using the 123 configuration, meaning a single sequence per species, per locus. We estimated parameters under the M0 model (null model) and M2 model (gene flow model). For the M0 model, divergence times for the root and for the in-group species are estimated alongside population sizes for the two ancestral populations, whilst M2 estimates four additional parameters including population sizes for the two extant in-group species and unidirectional gene flow parameters in both directions (A🡨B and A🡪B).

## Molecular evolution across the *virilis* group

Gene models produced by annotation were extracted from a representative genome for each species in the *virilis* group. Orthofinder was used to cluster orthologs (Emms and Kelly 2015, 2019). Multiple sequence alignments were extracted from Orthofinder output, and putative orthologs were filtered for paralogs by removing species with more than one sequence in every orthogroup. A custom script was used to retrieve corresponding nucleotide sequences based on amino acid alignments of orthologs produced by Orthofinder. Alignments were then filtered for length, removing alignments with sequences smaller than 150 nucleotides. Additionally, alignments with less than 8 species were filtered at this stage too. Alignments were then made using MAFFT with default options (Katoh and Standley 2013). Finally, alignments were trimmed using trimAl with the parameter ‘--gappyout’ (Capella-Gutiérrez et al. 2009). Species trees for each file were constructed for each alignment by pruning species from the species tree that were filtered from the alignments. Here, we first assessed d*N*/d*S* across the *virilis* group and for each alignment using the M0 model via ete3 (Yang et al. 2000; Huerta-Cepas et al. 2016).

Additionally, we used a clade-specific branch-site test to identify genes evolving rapidly on each of the three clades in the *virilis* group using a clade-specific branch-site test (bsC vs. M1)(Yang and Nielsen 2002). Models were compared using a likelihood ratio test and p-values were corrected for multiple testing strictly using false discovery rate (p < 0.01). To understand the function of significant genes we used Blast2GO (Conesa et al. 2005). To obtain more specific gene ontology predictions, we retrieved one representative sequence from the alignments of significant genes and blasted them against NCBI non-redundant database. Uniprot identifiers for best-hits were then converted into corresponding D.melanogaster orthologs, where possible, using Flymine (Lyne et al. 2007). Finally, we used FlyEnrichr for gene annotation and gene ontology prediction (Kuleshov et al. 2019).

# Supplementary Results

## Molecular evolution of protein-coding genes across the *virilis* group.

After correcting for multiple testing and filtering for saturation (dS > 2), we found 39 genes out of 7,443 genes with ω > 1 when calculating substitution rates across the entire *virilis* group (M0 model) (Supplementary figure 1 and Supplementary table 4. These included FASN2 (ω=1.08), a gene responsible for the production of methyl-branched cuticular hydrocarbons which contribute to reproductive isolation between *D. birchii* and *D. serrata* of the *montium* group (Chung et al. 2014) and are under sexual selection in *D. montana* (Veltsos et al. 2012; Jennings et al. 2014)*.* Genes involved in sensory perception (Dhc36a, Or2a and CheA7A) were also among those showing evidence for rapid adaptive sequence evolution across the *virilis* group.

We conducted phylad-specific branch-site tests, with branches in each phylad considered foreground branches with different rates of ω to background branches (bsC model vs. M1) (Supplementary figure 1A). We corrected for multiple testing (FDR=0.01) and filtered for saturation (dS > 2, dN/dS > 2), and detected 214 genes in the *littoralis* phylad, 278 genes in the *montana* phylad, and finally, 218 genes in the *virilis* phylad, that had significantly different branch-site ratios compared to background branches (Supplementary Table 5). We found 83 genes with significantly different branch-site ratios across all three phylad-specific tests. Between the *littoralis* and *montana* phylads, and between the *littoralis* phylad and the *virilis* phylad, we found an overlap of 47 and 33 genes respectively, with the *montana* phylad and the *virilis* phylad sharing an overlap of only 17 genes. Gene ontology inference of biological processes identified regulation of locomotor rhythm, antimicrobial response and regulation of triglyceride metabolic process.


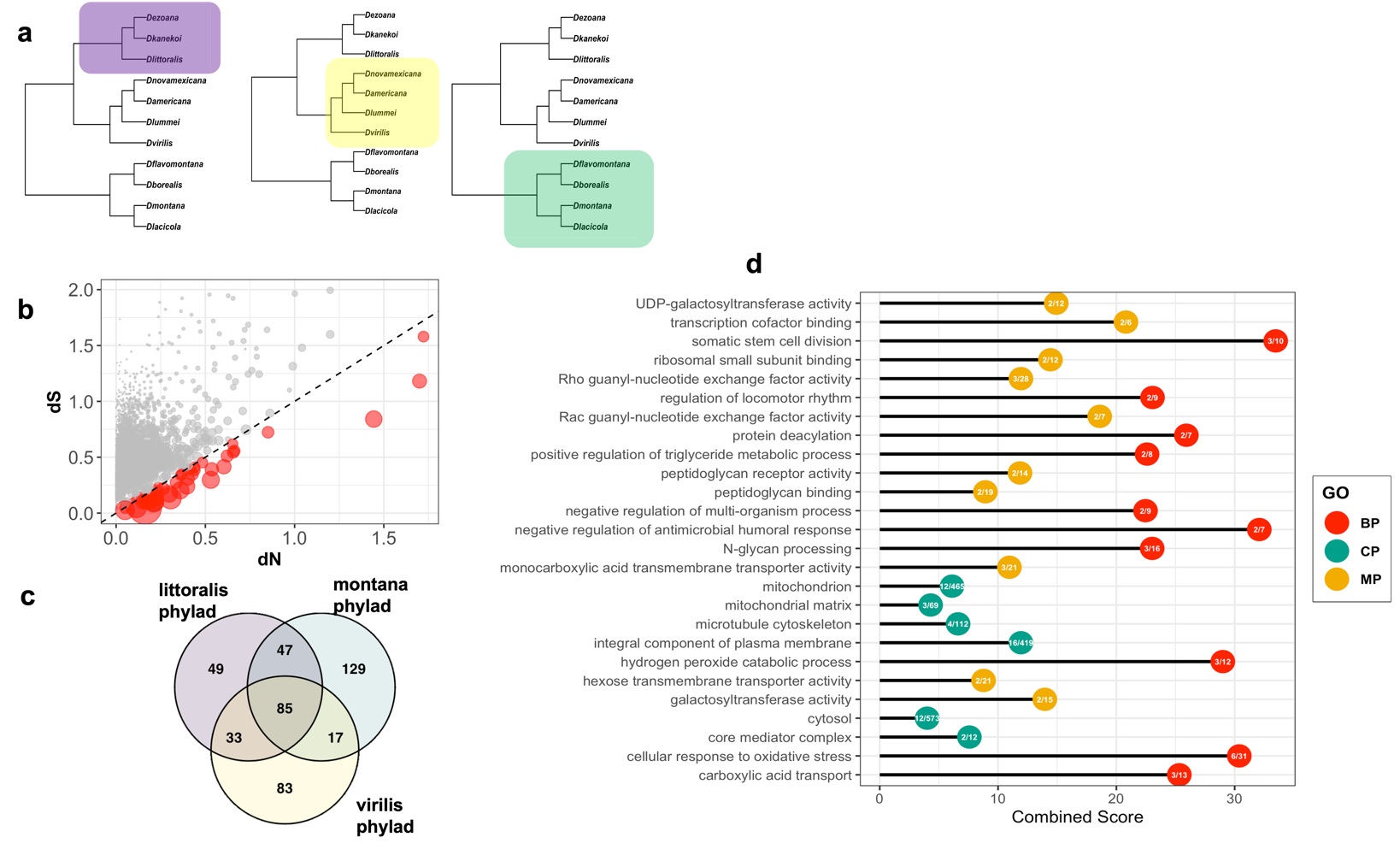


**Supplementary figure 1:** Detection of genes under putative positive selection in the virili*s* group. **a)** Illustration showing phylogenies and phylad tested in phylad-specific branch-site test using PAML. **b)** Synonymous substitution rate against non-synonymous substitution rate across entire gene tree for orthologs in the *virilis* group. Dashed line denotes dN/dS=1 and red circles are genes with dN/dS values exceeding 1. Size of circles corresponds to dN/dS ratio **c)** Overlap in genes found in branch-site test between the three phylads tested. **d)** Gene ontology for genes detected to be under selection during phylad-specific branch-site test. Gene ontology terms in legend are: Biological Process (BP); Cellular Process (CP) and Molecular Process (MP). Inside each circle is the number of genes under putative selection that overlap with the reference gene set for each gene ontology term. Only terms with a significant p-value included and the top ten ontology terms for each category shown (except for CP, where only 6 genes showed significant p-values).


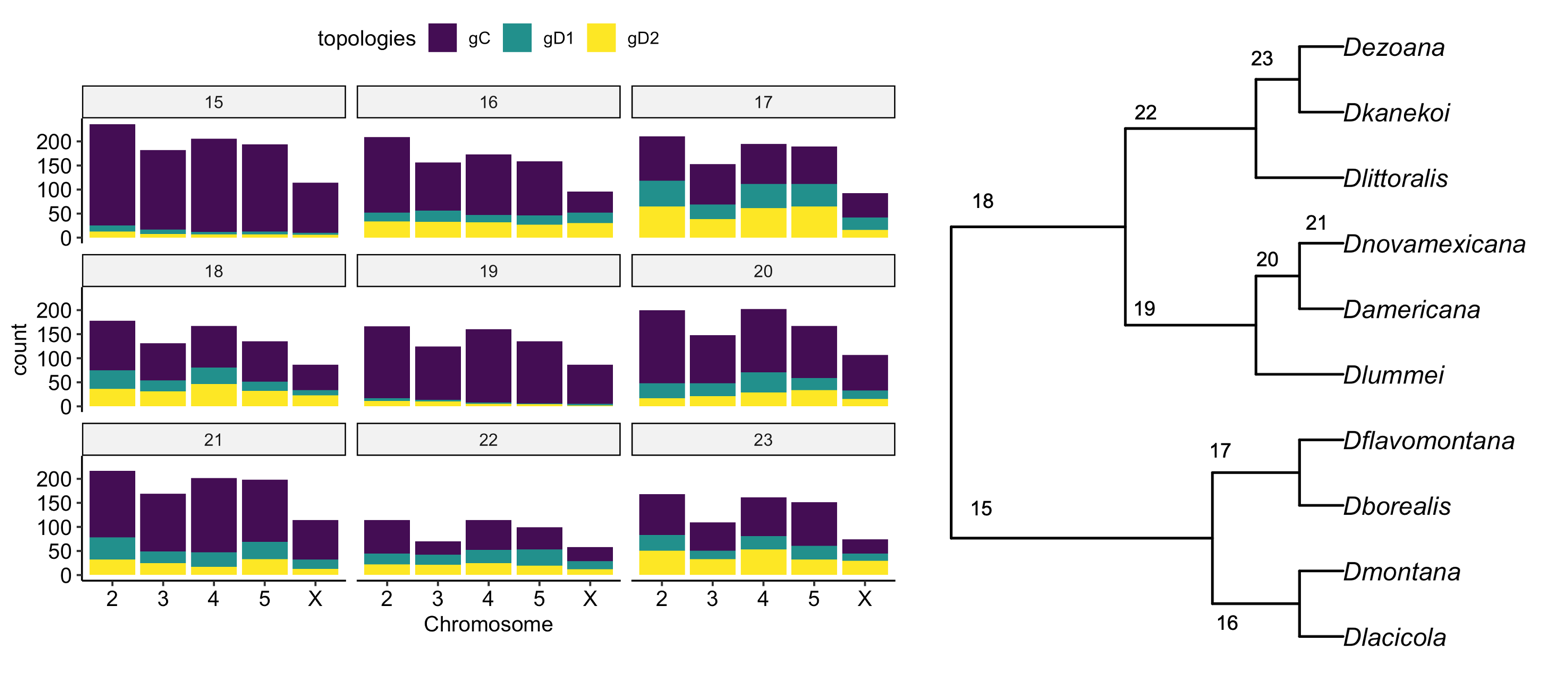


**Supplementary figure 2:** Gene concordance across nodes and chromosomes on the species tree. On the y-axis, counts denote the number of decisive gene trees supporting the species tree topology (gC; purple) and alternative topologies (gD1 and gD2; green and yellow). Number labels on the top of each plot show gene concordance for specific nodes on the species tree phylogeny (indicated on the left).


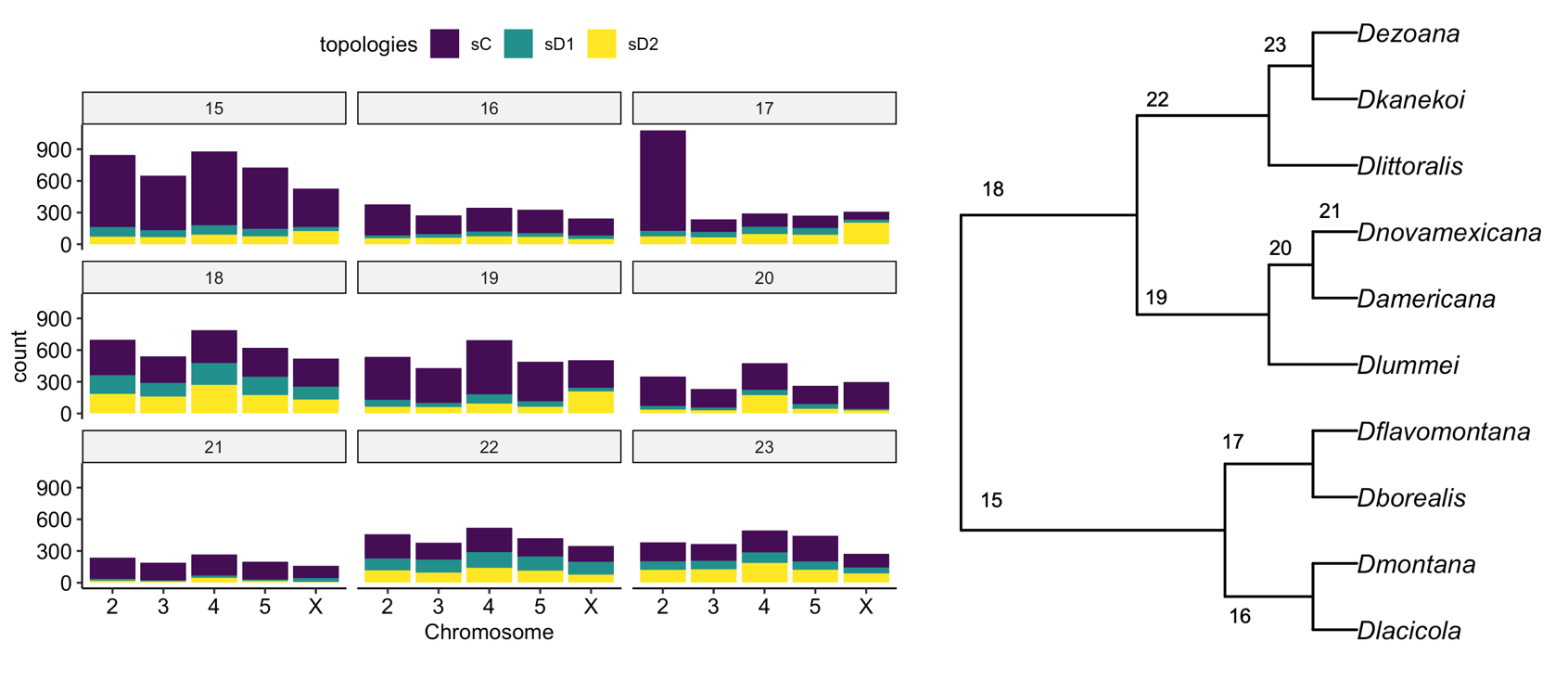


**Supplementary figure 3:** Site concordance across nodes and chromosomes on the species tree. On the y-axis, counts denote the number of decisive sites in the alignment supporting the species tree topology (sC; purple) and alternative topologies (sD1 and sD2; green and yellow). Number labels on the top of each plot show gene concordance for specific nodes on the species tree phylogeny (indicated on the left).


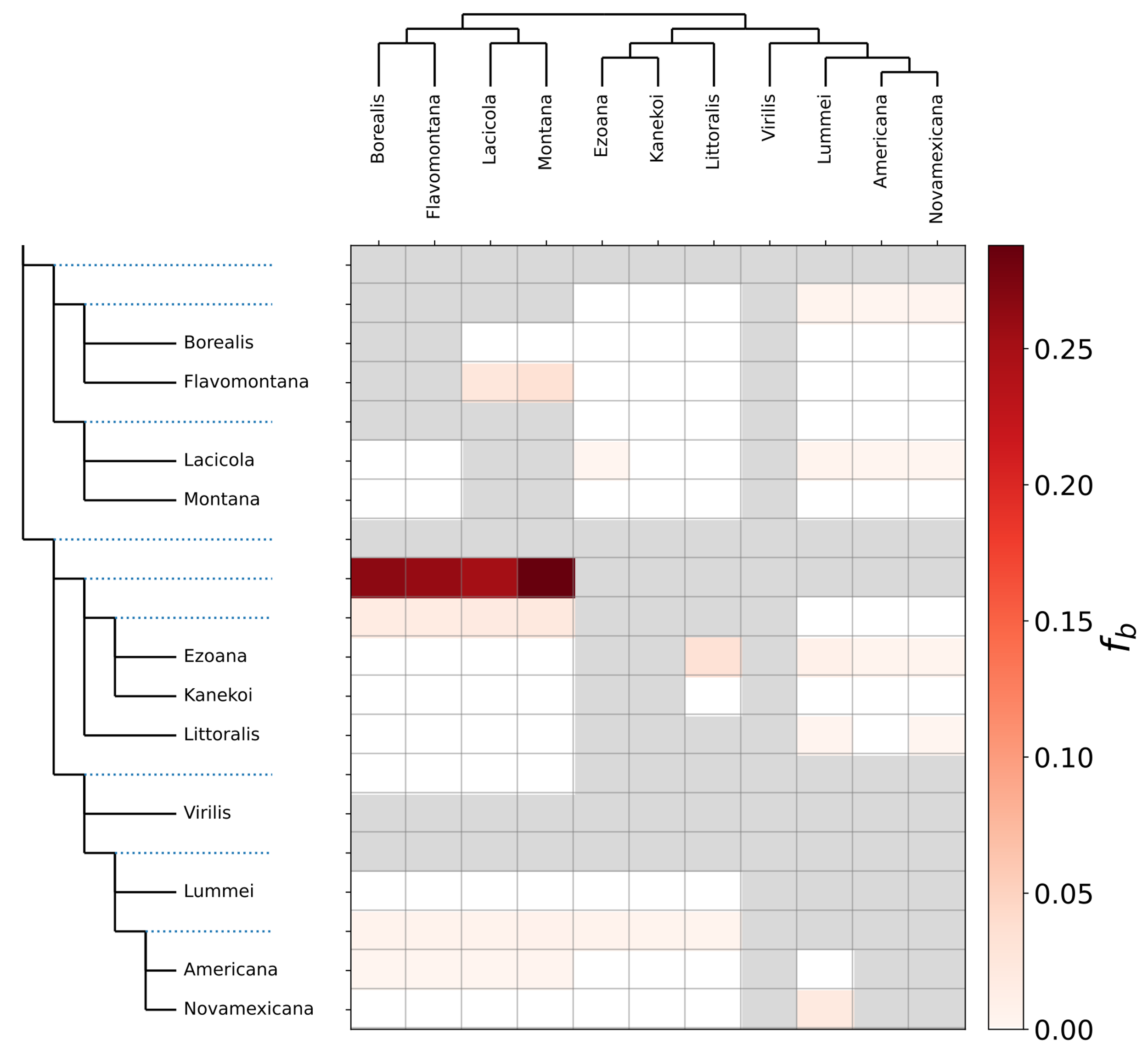


**Supplementary figure 4:** Summary of f-branch (f_b_) tests for introgression in the *virilis* group. Columns represent tips in the phylogeny, whilst rows represent nodes in the tree topology. Colours in cells denote f_b_ statistic between tree nodes and tree tips. Grey cells denote instances where comparisons could not be made.

**Supplementary figure 5:** Topology weighting shows widespread phylogenetic discordance across the virilis group. **a)** Topology weighting for each possible topology split by phylad, where green, purple and yellow violins show alternative topology weightings for the *montana* phylad, orange, red and brown violins show alternative topology weightings for the *littoralis* phylad, and pink, light blue and tan trees show alternative topology weightings for the *virilis* phylad. Topologies with values of 1 indicate topologies with maximal weighting. Topology weighting separately for each chromosome, where each row is a different chromosome (numbers to the left of violin plots denote chromosome). **b)** Topology each colour represents. Here, species names are abbreviated (M:*D. montana*, La: *D. lacicola*, B:*D. borealis*, E:*D. ezoana*, Li: *D. littoralis*, K:*D. kanekoi*, N:*D. novamexicana*, Lu :*D. lummei* and A:*D. americana.* Asterisks on topologies represent topologies that are concordant with the species tree.


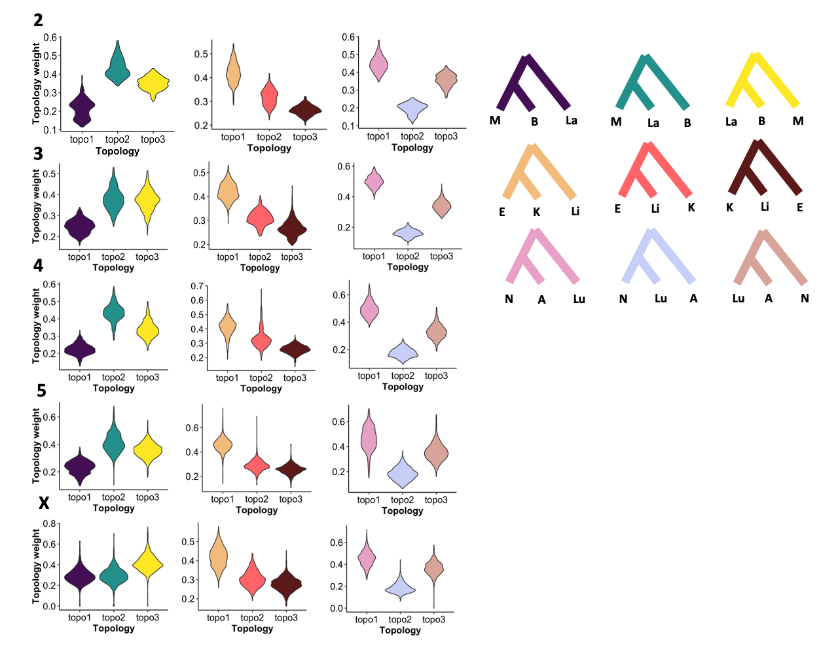


**a)**

**b)**

*

*

*


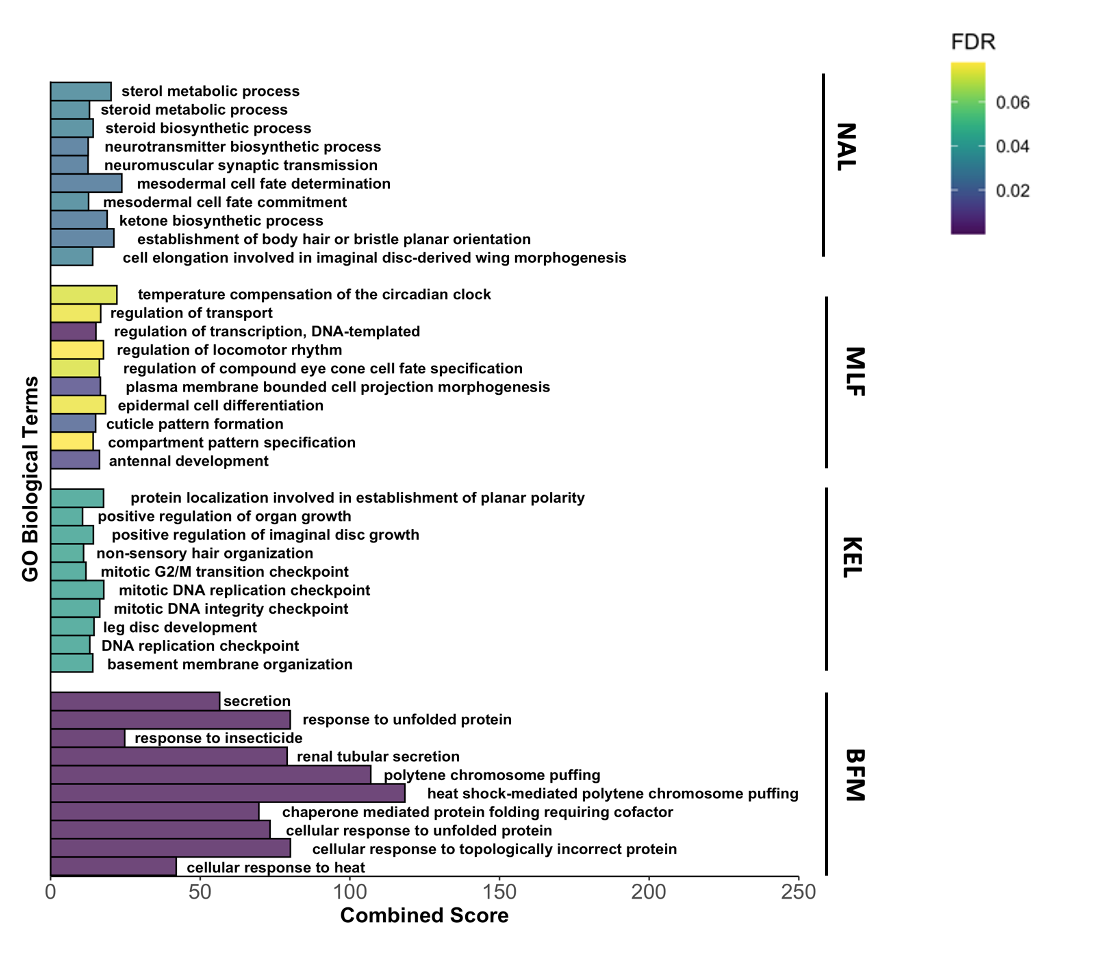


**Supplementary figure 6:** Gene ontology for genes showing signatures of admixture between trios. Here, we show gene ontology biological terms against combined score statistic, where the combined score statistic is calculated by multiplying the log of the p-value for the Fisher exact test by a Z-score computed by assessing deviation from an expected rank, using FlyEnrichr. Biological terms are split by trio, with NAL indicating gene flow from *D. lummei* into either *D. novamexicana* and *D. americana*, MLF indicating gene flow from *D. flavomontana* into *D. montana* and *D. lummei*, KEL indicating gene flow from *D. littoralis* into *D. ezoana* and *D. kanekoi*, and finally BFM indicating gene flow from *D. montana* into *D. flavomontana* and *D. borealis.*


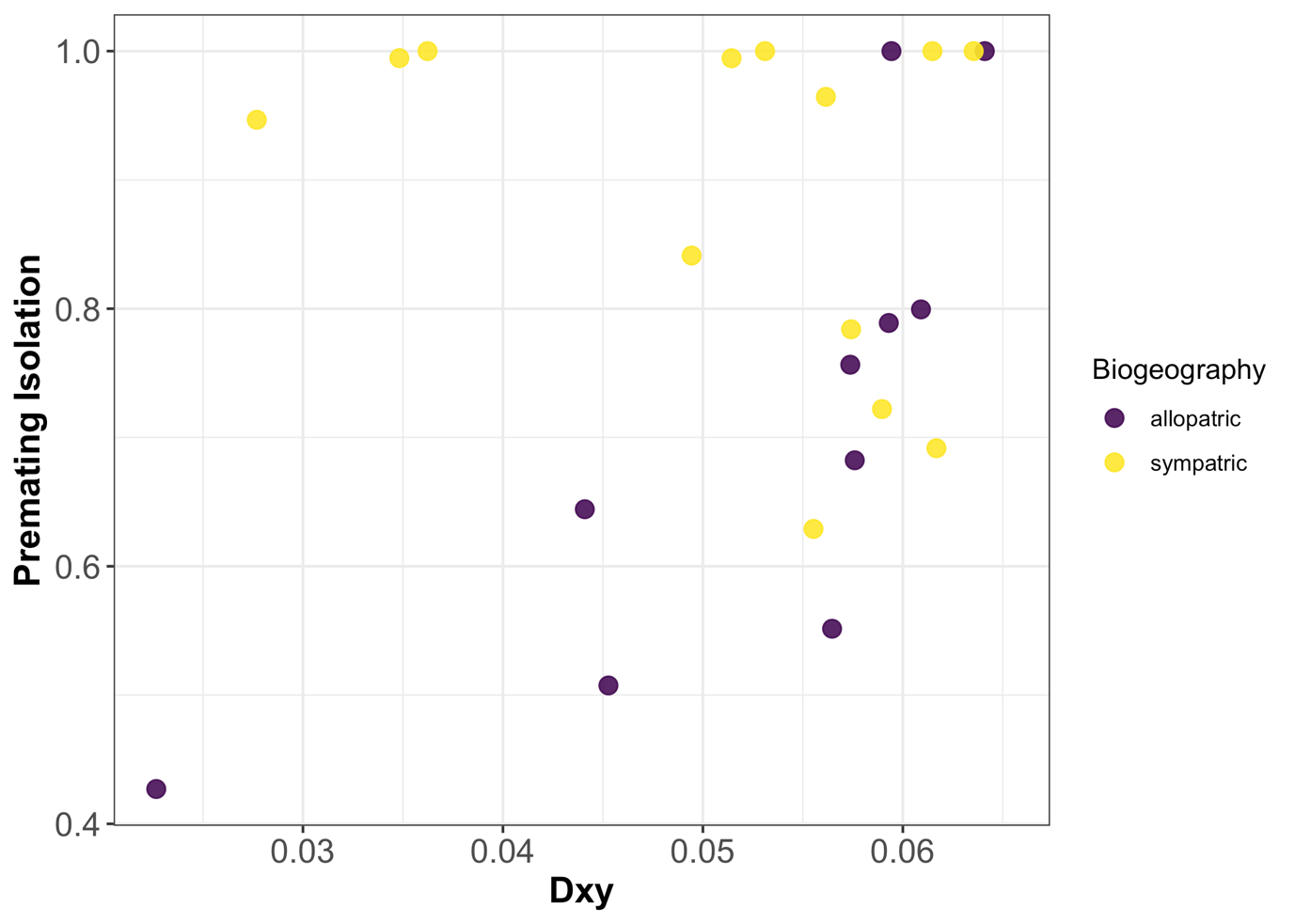


**Supplementary figure 7:** Absolute genetic divergence against pre-mating isolation for species pairs across the *virilis* group. Colours denote species pairs in allopatry (purple) and sympatry (yellow). Estimates of pre-mating isolation were taken from Yukilevich (2014) and originally estimated in Coyne and Orr (1989, 1997) and Throckmorton (1982) , with 1 denoting complete pre-mating isolation and vice versa.


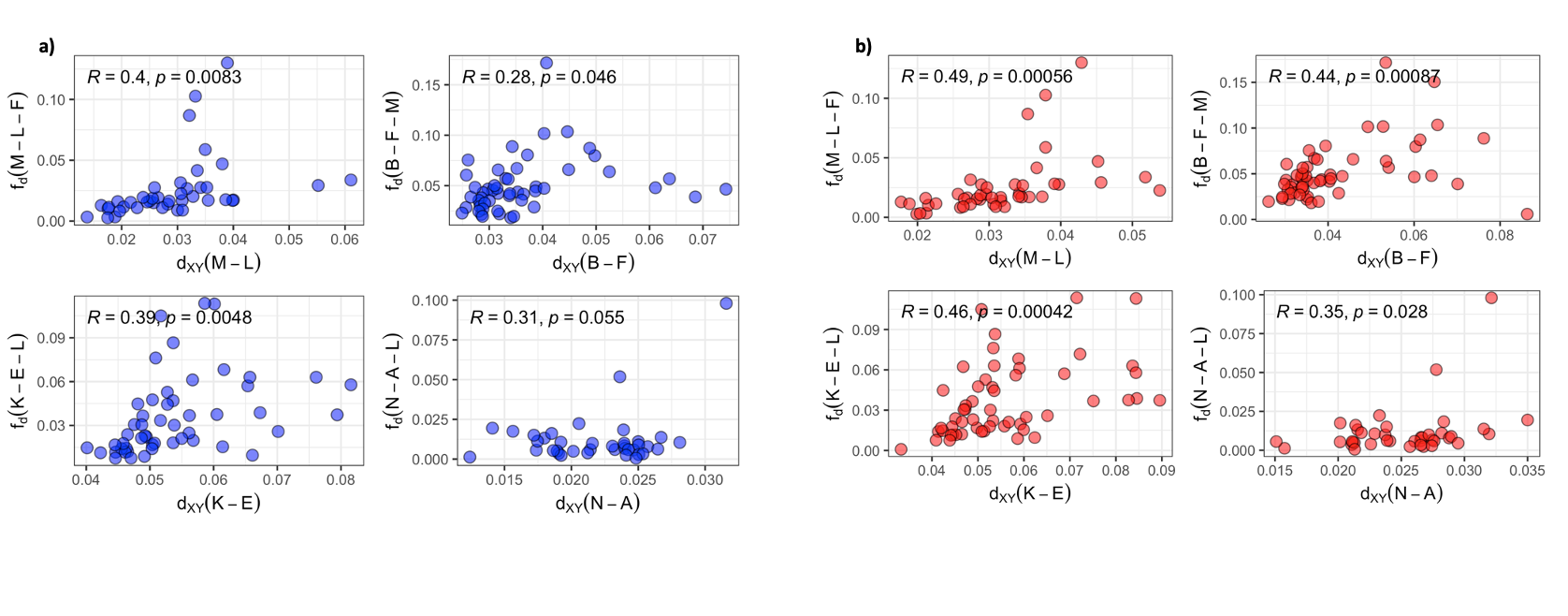


**Supplementary figure 8:** Mean admixture proportions for every scaffold was plotted for closely-related trios across the *virilis* group, against absolute genetic divergence in ‘true’ species pairs. **a)** Showing the correlation between admixture proportions (f_dm_) and genetic divergence in coding regions. **b)** Showing the correlation between admixture proportions (f_dm_) and genetic divergence in non-coding regions. For each plot, Pearson correlation coefficient was calculated. Here, species names are abbreviated (M: *D. montana*, La: *D. lacicola*, B: *D. borealis*, F: *D. flavomontana* E: *D. ezoana*, Li: *D. littoralis*, K: *D. kanekoi*, N: *D. novamexicana*, Lu: *D. lummei* and A: *D. americana.* Comparisons for admixture proportions are explained in Supplementary figure 6.
